# Perineal Groove: Report of Two Cases and Review of the Literature

**DOI:** 10.3389/fped.2018.00227

**Published:** 2018-08-15

**Authors:** Huiyan Cheng, Zhichao Wang, Qiang Zhao, He Zhu, Tianmin Xu

**Affiliations:** ^1^Department of Gynecology and Obstetrics, Second Hospital of Jilin University, Changchun, China; ^2^Department of Gynecology and Obstetrics, First Hospital of Jilin University, Changchun, China; ^3^Department of Pediatric Surgery, First Hospital of Jilin University, Changchun, China

**Keywords:** perineal groove, perineal raphe, congenital abnormality, neonatal, treatment

## Abstract

Perineal groove is a rare anoperineal congenital malformation disease that usually affects newborn females. It is unknown to many clinicians, which usually leads to misdiagnosis. The pathogenesis of perineal groove is not clear, and there are few cases reported in the current medical literature. Perineal grooves in two newborn babies were described in this report, and the literature on perineal groove was also reviewed and analyzed to improve the recognition of this disease.

## Introduction

Congenital perineal groove is rare, and few cases have been reported. A previous report published by Kadowai et al. (1) described perineal groove as an exposed erythematous nonepithelized mucous membrane extending from the vaginal fourchette to the anus (1). This condition is unknown to many obstetricians and pediatricians, and it is usually misdiagnosed as an anal fissure, perineal trauma, diaper dermatitis, infection, or sexual abuse (2). The incidence and pathogenesis of perineal groove are not clear. Appropriate counseling and follow-up can be provided by recognizing the disease of congenital perineal groove. This article describes two cases of congenital perineal groove and presents a review of the published literature on the epidemiology, pathogenesis, diagnosis, treatment, and prognosis of congenital perineal groove.

### Case one

**Figure 1:**
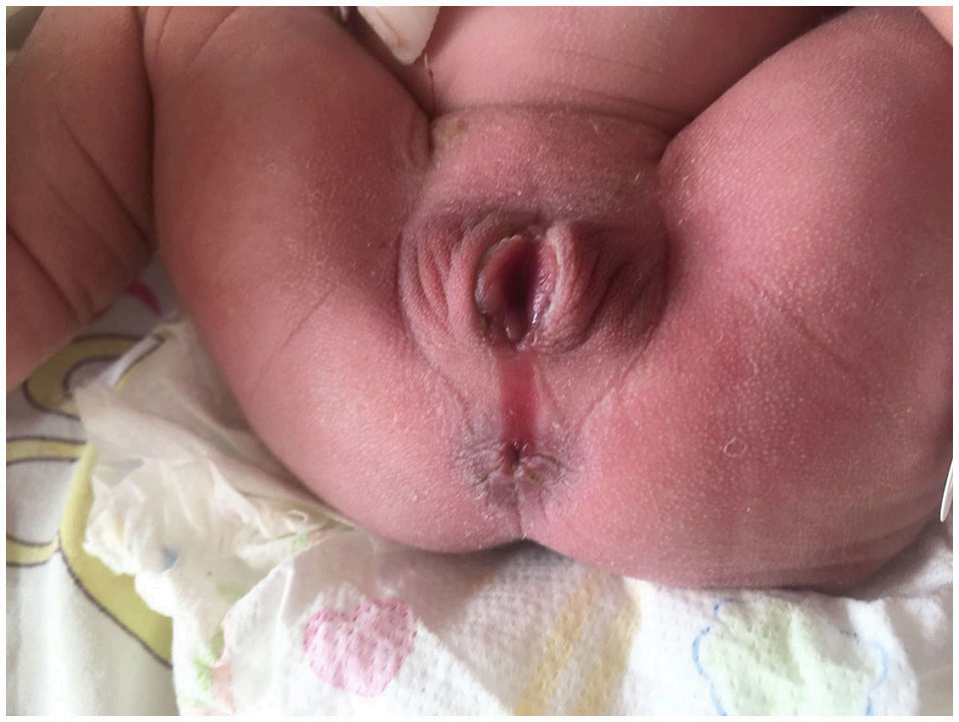
Wet groove between vulva and anus of case one.

This infant's parents were both of Asian descent. The mother was gravida 1 and para 0 (G1P0). The ages of the mother and father were 19 and 24, respectively, and they were both healthy. Neither of the infant's parents had a family history of congenital abnormalities, and they were not consanguineous. The mother had no history of tobacco, alcohol or substance abuse during the pregnancy. The father had no remarkable medical history. There were no other abnormalities during pregnancy. The infant's gestational age was 37 weeks and 5 days. Apgar scores were not clear when the infant was born. Owing to a birth weight (BW) of 1930 g, the newborn baby, who was delivered by normal spontaneous vaginal delivery, was admitted to the neonatal intensive care unit (NICU) for further examinations. Physical examination of the perineum revealed a wet groove-like erythematous nonepithelized mucous membrane extending vertically downward from the posterior vaginal fourchette to the anterior anal verge when the infant's legs were flexed. The lesion was ~2 cm from the base of vaginal fourchette to the anterior rim of anus at the 12 o'clock position. The width of the lesion was ~0.1 cm, and the depth of the lesion was ~0.1 cm. The surface of the perineal groove had no fistula, secretions, bleeding, or infection. The urethral canal, vagina and anus were in the appropriate position, and the anal wink was intact. The spine and sacral area had no obvious abnormalities (Figure 1). On admission, the infant had no problem with excretory functions. Her vital signs were as follows: temperature of 36.4°C; heart rate of 138 beats/min respiratory rate of 45 breaths/min; and blood pressure of 65/46 mmHg. Through a more thorough examination of the neonate, she was diagnosed with a low birth weight (LBW), neonatal wet lung disease, patent ductus arteriosus (PDA), intracerebral hemorrhage (ICH), neonatal polycythemia (NP), and congenital perineal groove. The diagnosis of perineal groove was made based on the clinical examination. The patient was recommended for genetic testing, but her parents declined. After 4 days of hospitalization, the conditions of the infant's other diseases had improved. The neonate was discharged.

### Case two

**Figure 2:**
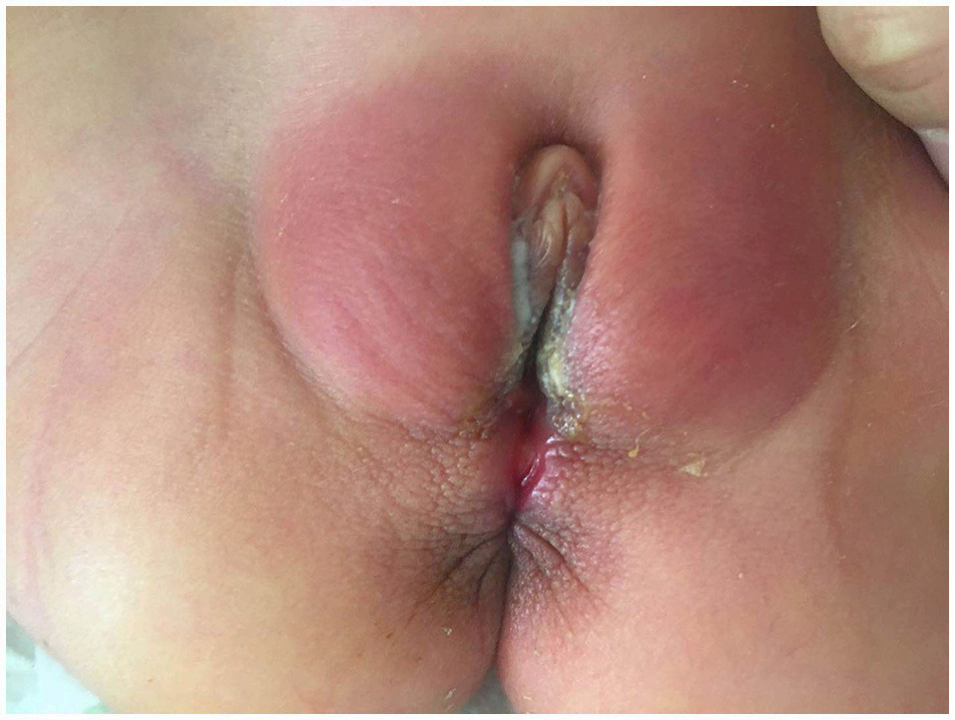
Wet groove between vulva and anus of case two.

This infant was born to Asian parents. The baby's mother was a 34-year-old woman, and her father was a 32-year-old man. The mother was gravida 2 and para 1 (G2P1) with good prenatal care. Their first baby was induced owing to intrauterine fetal death, and the details were unknown. There was no family history of congenital abnormalities on either side of the family, and the parents were not consanguineous. The mother also had no history of tobacco, alcohol, or substance abuse. The mother's antenatal examination was uneventful except for gestational diabetes. During pregnancy, the mother's blood glucose was maintained between 6.1 mmol/l and 8.1 mmol/l without any drug treatment. The infant's gestational age was 37 weeks and 3 days. The newborn was delivered by vaginal delivery and her birth weight (BW) was 4910 g. Apgar scores were 6 and 8 at 1 and 5 min, respectively. Owing to dyspnea 2 h after the birth, the neonate also received further examination in the NICU. On examination, a perineal defect was noted. The groove extended vertically downward from the base of the vaginal fourchette to the anterior rim of the anus at the 12 o'clock position. The perineal groove was a moist red sulcus that was ~1 cm long, 0.1 cm wide and 0.1 cm deep. There were no signs of malformation, bleeding, fistula, secretions, or infection noted in the genital area (Figure 2). During admission, the infant had normal excretory functions. Her vital signs were as follows: temperature of 36.5°C; heart rate of 110 beats/min; respiratory rate of 65 breaths/min; and blood pressure of 75/39 mmHg. The newborn was diagnosed with asphyxia neonatorum, neonatal wet lung disease, fetal macrosomia, cephalohematoma of newborn, PDA, myocardial injury, and congenital perineal groove and as a neonate of a diabetic mother after further examinations. This diagnosis of perineal groove was also based on clinical examination. The neonate was discharged home with her parents at 9 days of life when all her conditions improved. Both patients in case one and case two were advised to undergo follow-up examinations.

## Discussion

To our knowledge, no more than 25 studies have been reported on congenital perineal groove. The characteristics of our two cases are summarized in Table 1. Perineal groove is generally described as a wet sulcus extending from the posterior fourchette of the vagina to the anterior anus. There are three characteristics of congenital perineal groove: (1) a wet groove in the perineum between the fourchette and the anus; (2) normal vestibular structures, such as the urethra or vagina; and (3) hypertrophy of the minoral tails which course posteriorly around the perineum to converge at the anus or surround it (3). Female patients are more commonly affected by congenital perineal groove than males (4). Only 2 cases have been reported in male patients (5, 6). When this disease occurs in males, it is usually associated with hypospadias and a bifid scrotum (7). Owing to the few literature studies on congenital perineal groove, the incidence, epidemiology, and pathogenesis of congenital perineal grooves have been unclear until now.

**Table 1:** The characters of two perineal groove cases in this paper.

**Case**	**Sex**	**Complications**	**Physical examinations of the lesion**			**The characters of maternal**		
	**Weight**		**Fistula**	**Secretions**	**Size of lesion**	**Age**	**Complications**	**Gravidity and parity history**
One	female	Low birth weight infants(LBW)	No	No	long:2.0cm	19	No	G1P0
	1930g	Wet lung disease of neonatal			Wide: 0.1cm			(GA: 37 weeks and 5 days)
		Patent ductus arteriosus(PDA)			Depth:0.1cm			
		Intracerebral hemorrhage(ICH)						
Two	female	Asphyxia neonatorum	No	No	long:1.0cm	34	gestational diabetes	G2P1
	4910g	Wet lung disease of neonatal			Wide: 0.1cm			(GA: 37 weeks and 3 days)
		Fetal macrosomia			Depth:0.1cm			
		Cephalohematoma of newborn						
		Patent ductus arteriosus(PDA)						
		Myocardial injury						

*G, Gravidity; P, Parity; GA, Gestational age*.

There are several embryological hypotheses regarding the development of the perineal groove, which have been controversial until now. Defects in the development of the uroanal septum may cause perineal groove and perineal canal (8). The formation of perineal groove may be due to failure of fusion of the median genital folds, which are located on the midline (9). It has also been suggested that the formation of perineal groove could be due to be a remnant of the open cloacal duct (10). It has also been hypothesized that perineal groove and perineal canal occur as uroanal septal defects after the normal urorectal septum (URS) forms (3). The abnormal expression of SHH, Gli2, Gli3, Hoxa-13/Hoxd-13, Fgf10, and bone morphogenic protein 4 (BMP4) may be associated with congenital perineal groove. The parents of our two infants both declined genetic testing. Both of the infants suffered from PDA but had no other deformities of the urogenital canal or anus. The exact genetic changes and embryologic origins of congenital perineal groove are poorly understood and need further research. Table 1 demonstrates the following: (1) The age of the patient's mother in case one was 19, and the weight of the newborn was 1930 g. (2) The age of the patient's mother in case two was 34, and the weight of the newborn was 4910 g. (3) The mother of the patient in case two had gestational diabetes, as reported by Diaz (4) and Sekaran (8). Some patients with perineal defects at birth have mothers with mild thalassemia anemia (7), preeclampsia (11), placenta previa (11), group B streptococcal infections (12), etc. The age of the pregnant woman, the conditions of the newborn and maternal complications may be risk factors for congenital perineal groove.

The diagnosis of perineal groove mainly depends on clinical examination. However, there are no specific guidelines for classification. Through observation of perineal grooves in 7 patients, Shen et al. (13) described that perineal groove could be divided into complete perineal groove and incomplete perineal groove. Complete perineal groove is defined as a lesion extending from the vaginal opening to the anal sphincter. In addition, there were two types of incomplete perineal groove described in their study including higher incomplete perineal groove (from the vagina to the middle of the perineum but not to the anus) and lower incomplete perineal groove (from the anus to the middle of the perineum but not to the vagina).

In most patients, the lesions are asymptomatic, and they spontaneously epithelialize by ~1 year of age (1, 8). Conservative treatment is preferred. Surgical treatment is generally advised in the following situations: (1) for cosmetic reasons (11); (2) if epithelialization fails to occur by 2 years of age (the potential time for self-healing has elapsed) (14); or (3) if the groove is causing recurrent problems, such as infections, mucus drainage, or wet secretion from the urethra, vagina, or anal sites (4, 8). Some studies have shown that covering the suture line with a chemical glue results in perfect cicatrization after surgical treatment (14). Histological pathology of the perineal groove after surgery mostly demonstrates squamous epithelium (13). We reviewed 18 studies in Table 2. Most patients in these studies did not undergo surgery and healed with no complications in ~ 1 or 2 years. Therefore, long-term follow-up is essential for congenital perineal groove. This lesion is often misdiagnosed at birth if it cannot be correctly identified. This condition is different from other low rectal-anal deformities, such as rectal perineal fistula and cutaneous fistula, which require surgery. These low rectal-anal deformities are often associated with congenital anal atresia showing no anus at proctodeum. For rectal perineal fistula, there is a small crack in the midline of the perineum, and a small amount of meconium will be discharged from the crack. Cutaneous fistula can be seen as white or pigmented fistula with thin skin covering on the surface in the middle line of the perineum. Therefore, it is important to first determine whether there is an anorectal malformation that requires surgical treatment. After all, for perineal groove, unnecessary procedures and interventions are potentially invasive. The prognosis of perineal groove is mostly good.

**Table 2:** The case reports of literature review with the perineal groove.

**Author(s), Year**	**Cases in article(sex)**	**Age at the diagnosis**	**Treatment**	**Histology**	**Longest follow-up**	**Outcome(complications)**
Garcia-Palacios et al. (15)	2 (female)	Mean14M	No	NR	1Y	Partially epithelized (No)
Harsono and Pourcyrous. (11)	2 (female)	Newborn	No	NR	4M/1Y	NR (No)/ Healing (No)
Hunt and Srinivas. (12)	1 (female)	Newborn	No	NR	6M	Healed (No)
Barbosa et al,2016 (16)	1 (female)	Newborn	No	NR	6M	Healed (No)
Senanayake andTennakoon. (2)	1 (female)	26M	No	NR	at yearly	Healed (No)
Pastene and Rojas. (17)	2 (female)	4M/8Y	No	NR	4M/NR	Clinic (No)
Shen W et al. (18)	7 (female)	Mean 3Y	No (3)/surgery (4)	NR	1Y to 4Y	All healed (No)
Diaz et al. (4)	2 (female)	4M /6M	No	NR	12M/8M	Remain stable (No)/ less pronounce (No)
Siruguppa et al. (7)	1 (female)	Newborn	No	NR	1M	Healing (No)
Carrera Polanco (19)	1 (female)	2.5Y	No	NR	NR	NR (NR)
Esposito et al. (14)	6 (female)	Mean 4.5 Y	Surgery	Squamous	long-term	2 dehiscences/ 4Healed (No)
Verma and Wollina (20)	1 (female)	2.5 Y	No	NR	NR	NR (NR)
Sekaran and Shawis. (8)	1 (female)	Newborn	No	NR	Yearly	Healed (No)
Mullassery et al. (13)	1 (female)	6 M	Surgery	Squamous	NR	NR (NR)
Aslan et al. (6)	1 (male)	Newborn	Surgery	Squamous epithelium	21M	Not Healed
Chatterjee et al. (5)	1 (male)	7 Y	Surgery	Nonkeratinizing,squamous	2Y	Healed (No)
Kadowaki et al. (1)	2 (female)	2 M /3 M	No/surgery	Squamous	6Y/14M	Healed (No)
F. Douglas Stephens. (3)	4 (female)	NR	NR	NR	NR	NR

*NR, Not reported; M, Months; Y, Years; D, Days*.

## Conclusion

The incidence of congenital perineal groove is low, and the incidence in women is higher than that in men. Most cases tend to self-epithelialize over time (~1 or 2 years). The diagnosis of congenital perineal groove depends on clinical examination. The pathogenesis is unknown. Conservative treatment is generally preferred for congenital perineal groove. Confusion regarding the diagnosis may lead to misdiagnosis and unnecessary surgical or medical intervention, and we report two cases of perineal groove to raise awareness of this unusual malformation. Understanding perineal groove as an abnormal perineum will help to avoid misdiagnosis and prevent excessive interventions or unnecessary surgical procedures. At the same time, as doctors, we can provide appropriate advice and follow-up recommendations to the parents of affected infants.

## Ethics approval and consent to participate

This study was approved by the Ethical Review Committee of The First Hospital of Jilin University.

## Author contributions

HC drafted the manuscript. ZW diagnosed the malformation and revised the manuscript. HC and ZW contributed equally to this article and should be considered co-first authors. QZ and HZ diagnosed the malformation and provided advice regarding the manuscript. TX designed the topic and revised the manuscript. All authors read and approved the final manuscript. All authors agree to be accountable for the content of the work.

### Conflict of interest statement

The authors declare that the research was conducted in the absence of any commercial or financial relationships that could be construed as a potential conflict of interest.
